# Renal resistive index as an indicator of the presence and severity of anemia and its future development in patients with hypertension

**DOI:** 10.1186/s12882-015-0040-6

**Published:** 2015-04-08

**Authors:** Muneyoshi Tanimura, Kaoru Dohi, Masumi Matsuda, Yuichi Sato, Emiyo Sugiura, Naoto Kumagai, Shiro Nakamori, Tomomi Yamada, Naoki Fujimoto, Takashi Tanigawa, Norikazu Yamada, Mashio Nakamura, Masaaki Ito

**Affiliations:** Department of Cardiology and Nephrology, Mie University Graduate School of Medicine, Tsu, Japan; Central Clinical Laboratories, Mie University Hospital, Tsu, Japan; Department of Translational Medical Science, Mie University Graduate School of Medicine, Tsu, Japan; Department of Molecular and Laboratory Medicine, Mie University Graduate School of Medicine, Tsu, Japan

**Keywords:** Chronic kidney disease, Hypertension, Renal resistive index, Anemia

## Abstract

**Background:**

We examined whether renal resistive index (RI), a simple index of renal vascular resistance, is associated with the presence and severity of anemia, and can predict the future development of anemia in patients with hypertension.

**Methods:**

We retrospectively examined 175 patients with hypertension (mean age 67 ± 11 years, 32-85 years, 134 males) who underwent renal ultrasonography. Anemia was defined as a reduction in the concentration of hemoglobin <13.0 g/dL for men and <12.0 g/dL for women. Renal RI was measured in the interlobar arteries.

**Results:**

Anemia was present in 37% of men and 34% of women. The mean estimated glomerular filtration rate (eGFR) was 58 ± 23 ml/min/1.73 m^2^ (median: 56 ml/min/1.73 m^2^, range: 16-168 ml/min/1.73 m^2^) and the mean renal RI was 0.70 ± 0.09 (median: 0.70, range: 0.45-0.92). Proteinuria was present in 29% of patients. Both eGFR and renal RI correlated significantly with hemoglobin levels. In the stepwise multivariate linear regression analysis, renal RI was associated with hemoglobin levels independently of potential confounders including eGFR. During the follow-up period (median: 959 days, range: 7-3595 days), Kaplan–Meier curves demonstrated that patients with renal RI above the median value had a higher incidence of the future development of anemia than other patients. Cox regression analysis showed that renal RI (hazard ratio 1.18, 95% CI 1.02-1.37 per 0.05 rises in renal RI, p =0.03) and the presence of proteinuria were (hazard ratio 1.80, 95% CI 1.08-3.01, p =0.03) were independently associated with the future development of anemia after correcting for confounding factors.

**Conclusions:**

Measurement of renal RI can be useful for elucidating the pathogenesis of anemia and for inferring its potential risk in patients with hypertension.

## Background

Chronic kidney disease (CKD) is known to cause anemia mainly due to inappropriate erythropoietin (EPO) secretion [1,2], especially in patients with end-stage renal failure. However, anemia can occur early in the development of CKD, defined on the basis of the estimated glomerular filtration rate (eGFR). This indicates that the progression of renal anemia is not governed solely by glomerular function [3]. In recent studies, EPO-producing cells were identified in the renal tubulointerstitium surrounding the central vessels, not in the glomeruli [4,5]. Thus, unfavorable changes in the interstitial microenvironment, such as inflammation, oxidative stress and ischemia, can impair the function of EPO-producing cells. Recently, Souma et al. demonstrated that the phenotypic transition of EPO-producing cells to non-EPO-producing myofibroblasts is modulated by inflammatory molecules, and suggested the connection between anemia and renal fibrosis in CKD in a mouse model [6].

Hypertension is one of the leading causes of CKD associated with arteriosclerosis, interstitial inflammation and fibrosis, as well as tubular insufficiency secondary to endothelial dysfunction and progressive infiltration of macrophages and T cells to the perivascular interstitium induced by chronic exposure to high blood pressure [7-10]. Therefore, the development of renal damage can contribute to the pathogenesis of anemia, even in earlier stages of CKD in patients with hypertension.

Renal resistive index (RI) in the intra-renal arteries at the level of the corticomedullary junction using pulsed Doppler ultrasonography is a simple index of renal vascular resistance [11,12], and high renal RI is known to be associated with severe interstitial fibrosis, arteriosclerosis and renal function decline [13-15].

Accordingly, we examined whether renal RI is associated with the presence and severity of anemia, and whether high renal RI predicts the future development of anemia in patients with hypertension.

## Methods

### Study population

We retrospectively reviewed patients with hypertension who underwent renal ultrasonography for the screening of renal artery stenosis and the evaluation of renal arteriosclerosis at their physician’s discretion on the basis of their age, comorbidity and disease characteristics in Mie University Hospital between April 2004 and June 2012. Among them, we selected 175 subjects (mean age 67 ± 11 years, range 32-85 years, 134 males) after excluding patients with atrial fibrillation, moderate to severe aortic valvular heart diseases, congestive heart failure, primary glomerular and tubulointerstitial disease of the kidney, hydronephrosis, nephrotic syndrome, renal artery stenosis, and diseases or conditions that could cause or improve anemia (such as advanced cancers, hematologic disorders, autoimmune diseases and active bleeding). Patients receiving hemodialysis or erythropoiesis-stimulating agent therapy were also excluded.

### Clinical evaluation

Anemia was defined as a reduction in the concentration of hemoglobin in a sample of venous blood when compared with reference values (<12.0 g/dl for women and <13.0 g/dl for men) [16]. CKD was defined as the presence of eGFR <60 ml/min/1.73 m^2^ for 3 months or more. The eGFR of each patient was calculated from their serum creatinine (SCr) value and their age using the following equation: eGFR (ml/min/1.73 m^2^) = 194 × Age^-0.287^ × SCr^-1.094^ (if female × 0.739) [17]. Urine proteins were qualified by using a dipstick test. All patients were evaluated for the presence of coexisting traditional risk factors for atherosclerosis. Diabetes mellitus was determined under the following conditions: fasting glucose level >125 mg/dl, casual plasma glucose concentration >200 mg/dl in the presence of symptoms, 2-hour oral glucose tolerance test value of >200 mg/dl or if the patient was using antidiabetic agents, including insulin [18]. Hypertension was determined under the following conditions: systolic blood pressure ≥140 mmHg or diastolic blood pressure ≥90 mmHg, or if the patient was receiving antihypertensive therapy [19]. Dyslipidemia was determined under the following conditions: low-density-lipoprotein cholesterol level ≥140 mg/dl, triglyceride level ≥150 mg/dl, high-density-lipoprotein cholesterol level <40 mg/dl or receiving antidyslipidemic medication [20]. Finally, chronic obstructive pulmonary disease (COPD) was diagnosed on the basis of a post-bronchodilator forced expiratory volume in one second (FEV1)/forced vital capacity (FVC) value of <70 [21]. The protocol was approved by the Human Studies Subcommittee of Mie University Graduate School of Medicine.

### Renal doppler ultrasonography

**Figure 1:**
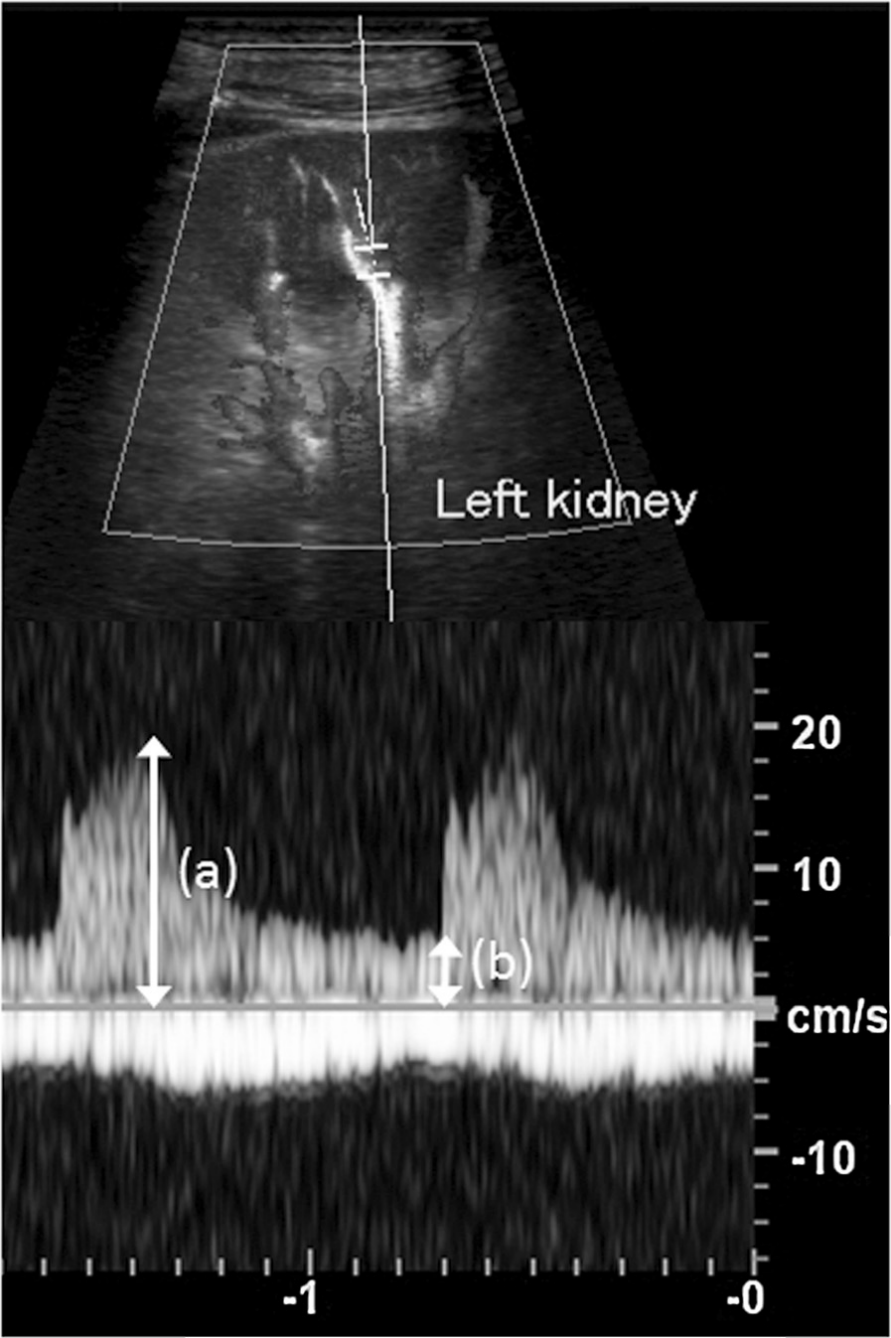
**Example of renal resistive index measurement.** Pulsed Doppler ultrasonography was obtained in the intra-renal artery at the level of the corticomedullary junction. Renal resistive index is measured as {peak systolic velocity **(a)** - end diastolic velocity **(b)**} / peak systolic velocity **(a)**.

First, the flow velocities in the aorta and renal arteries were evaluated to rule out morphological abnormality or renal artery stenosis. Second, the renal RI was determined in the interlobar arteries of both kidneys and expressed as the mean of these values. The digital diagnostic ultrasound systems used were the Aplio XG SSA-790A with a PVT-375BT convex array transducer (Toshiba Medical Systems, Otawara, Tochigi, Japan) operating at a frequency of 3.5 MHz, and the LOGIQ P6 with a 4C convex array transducer (GE Medical Systems, Milwaukee, WI, US) operating in the frequency range of 4.0 MHz to 5.5 MHz. The ultrasound examinations were performed by two well-trained technicians. Renal RI was calculated as follows (Figure 1):

RenalRI = (peak systolic velocity − end diastolic velocity)/peak systolic velocity [22,23].

## Clinical outcome

Medical records were retrospectively reviewed for each patient. The study end-point was 1) new anemia (<12.0 g/dl for women and <13.0 g/dl for men) for non-anemic patients, and 2) decreased hemoglobin levels greater than 1 g/dl and/or initiation of treatment including iron supplementation and EPO-stimulating agents for anemic patients. Anemic events within 3 months after minor surgery or cardiac catheterization and within 6 months after major surgery were excluded from the analysis.

### Statistical analysis

All continuous variables were approximately normally distributed and presented as the mean ± standard deviation. In order to assess differences between the two groups, Pearson’s chi-square test was used for nominal scales and Student’s t test was used for all other scales. To make comparisons among three groups, we used Bonferroni’s multivariate comparison test. The linear correlations between the variables were parametrically evaluated using Pearson’s product moment correlation coefficient. We performed stepwise multivariate linear regression analysis to determine independent predictors of hemoglobin levels. Moreover, we examined the predictive value of potential clinical parameters for the study end-point, namely, the future development of anemia. Cumulative event rates were assessed using the Kaplan–Meier method and compared using the log-rank test. The impact of clinical predictors on the future development of anemia was assessed by univariate and multivariate Cox proportional hazards regression. Hazard ratios are given with 95% confidence intervals (CI). Reliability was assessed by calculating intraobserver and interobserver intraclass correlation coefficients. For all tests, a p value <0.05 was considered statistically significant. Data were analyzed using SPSS Statistics for Windows (Version 20.0, IBM Corp., Armonk, NY, US).

## Results

### Patient characteristics

Patient characteristics are presented in Table 1. Hemoglobin level ranged from 7.3 to 17.2 g/dL, and anemia was present in 37% of men and 34% of women. Among all 175 patients, eGFR ranged from 16 to 168 ml/min/1.73 m^2^, and 102 patients (58%) had an eGFR of less than 60 ml/min/1.73 m^2^. Only 19 patients (11%) had an eGFR of less than 30 ml/min/1.73 m^2^. Fifty-one patients (29%) had a positive reaction in the urine protein test. The prevalence rates of coexisting diabetes mellitus, dyslipidemia and coronary artery diseases were 52%, 70% and 59%, respectively. When all subjects were divided into two groups according to the presence of anemia (Table 1), those with anemia were older, had greater body mass index (BMI), lower diastolic blood pressure, higher pulse pressure, higher renal RI, lower eGFR and higher prevalence of proteinuria than those without it. Patients with anemia were also more likely to receive renin-angiotensin-aldosterone system (RAAS) inhibitors and calcium channel blockers than those without it. There were statistically significant positive correlations between renal RI and pulse pressure, one of the surrogate measures of large artery stiffness (r = 0.45, p < 0.05), and age (r = 0.42, p < 0.05).

**Table 1:** **Patient characteristics and comparison between patients with and without anemia**

	**All**	**Anemia**	**No anemia**	**p value**
Demographic data				
Number	175	64	111	
Age, years	67 ± 11	71 ± 10	65 ± 11	<0.01
Males, number (%)	134 (76.6)	50 (78.1)	84 (75.7)	0.71
Body mass index, kg/m^2^	24.2 ± 3.9	23.7 ± 4.5	24.6 ± 3.5	0.02
Systolic blood pressure, mmHg	135 ± 18	137 ± 20	135 ± 17	0.59
Diastolic blood pressure, mmHg	75 ± 13	72 ± 14	76 ± 13	0.01
Pulse pressure, mmHg	61 ± 15	64 ± 17	59 ± 14	0.02
Heart rate, bpm	67 ± 11	67 ± 10	67 ± 12	0.98
Resistive index	0.70 ± 0.09	0.74 ± 0.09	0.67 ± 0.08	<0.01
Left ventricular ejection fraction	0.63 ± 0.12	0.63 ± 0.14	0.64 ± 0.11	0.92
Laboratory data				
Hemoglobin, g/dl	13.1 ± 1.7	11.4 ± 1.0	14.1 ± 1.2	<0.01
Estimated GFR, ml/min./1.73 m^2^	57.7 ± 23.1	48.3 ± 24.8	63.1 ± 20.3	<0.01
Qualitative urine protein, number (%)	51 (29.3)	31 (49.2)	20 (18.0)	<0.01
Hemoglobin A1c, %	6.4 ± 1.7	6.6 ± 2.1	6.4 ± 1.4	0.98
Comorbidities, number (%)				
Diabetes mellitus	91 (52.0)	35 (54.7)	56 (50.5)	0.60
Dyslipidemia	122 (69.7)	46 (71.9)	76 (68.5)	0.64
Coronary artery disease	104 (59.4)	41 (64.1)	63 (56.8)	0.34
Peripheral artery disease	34 (19.4)	16 (25.0)	18 (16.2)	0.16
History of congestive heart failure	12 (6.8)	6 (9.4)	6 (5.4)	0.35
History of cerebral infarction	26 (14.9)	12 (18.8)	14 (12.6)	0.30
Current smoking	53 (30.6)	19 (30.2)	34 (30.9)	0.92
COPD	8 (4.6)	4 (3.6)	4 (6.3)	0.41
Medications, number (%)				
RAAS inhibitors	125 (71.4)	52 (81.3)	73 (65.8)	0.02
Calcium channel blockers	114 (65.1)	47 (73.4)	67 (60.4)	0.07
Beta blockers	57 (32.6)	17 (26.6)	40 (36.0)	0.20
Statins	92 (52.6)	37 (57.8)	55 (49.5)	0.29

Values are expressed as mean ± SD or numbers and percentages. Student’s t test was used to assess differences between the two groups except for sex, qualitative urine protein, comorbidities and medications, for which chi-square test was used.

GFR, glomerular filtration rate; COPD, chronic obstructive pulmonary disease; RAAS, renin-angiotensin-aldosterone system.

### The relationships of eGFR and Renal RI to hemoglobin levels

**Figure 2:**
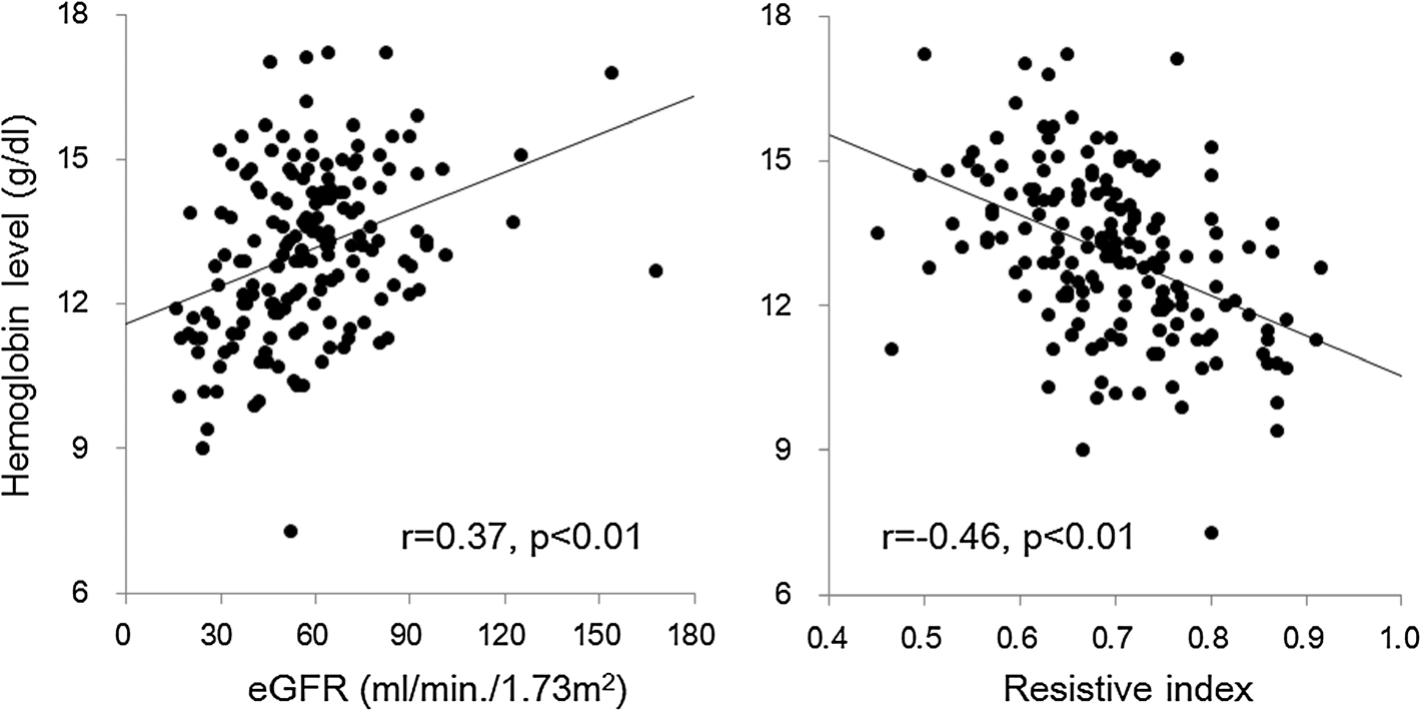
**The relationships between hemoglobin levels and estimated glomerular filtration rate and renal resistive index.** Scatter plots showing the relationships between hemoglobin levels and estimated glomerular filtration rate (left) and renal resistive index (right) in all 175 subjects. GFR, glomerular filtration rate.

**Figure 3:**
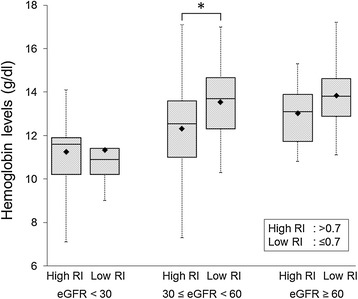
**Box and whisker plots showing the relationship between renal resistive index and hemoglobin level in each stage of CKD.** RI, resistive index; GFR, glomerular filtration rate.

All patients underwent successful renal Doppler ultrasonography, and the mean renal RI was 0.70 ± 0.09 (median: 0.70, range: 0.45-0.92). The measurements of renal RI were reproducible with intraobserver and interobserver intraclass correlation coefficients of 0.96 (p < 0.01) and 0.90 (p < 0.01), respectively. Figure 2 shows the relationships of renal RI and eGFR with hemoglobin levels. Both renal RI and eGFR had statistically significant correlations with hemoglobin levels. Further analysis revealed that there were significant correlations between renal RI and hemoglobin levels in the subgroups of 30 ≤ eGFR <60 ml/min/1.73 m^2^ (r = -0.42, p < 0.05) and eGFR ≥60 ml/min/1.73 m^2^ (r = -0.24, p < 0.05) but not eGFR <30 ml/min/1.73 m^2^ (r = -0.11, p = ns). In addition, we assessed the relationship between renal RI and hemoglobin level in each stage of CKD with box and whisker plots (Figure 3). Bonferroni’s multivariate comparison test showed that there was a significant difference in the hemoglobin levels between patients with renal RI higher than the median value (0.70) and those with renal RI ≤0.7 only in the subgroup of 30 ≤ eGFR <60 ml/min/1.73 m^2^. Subsequently, univariate and stepwise multivariate linear regression analyses were applied to assess the relationships between hemoglobin levels and clinical variables (p < 0.1 for entry in Table 1: age, sex, BMI, diastolic blood pressure, pulse pressure, renal RI, eGFR, urine protein and administration of RAAS inhibitors and calcium channel blockers). As a result, renal RI, eGFR, sex and BMI were independently associated with hemoglobin level (Table 2).

**Table 2:** **Univariate and stepwise multivariate regression of factors that correlate with hemoglobin levels**

	**Univariate**		**Multivariate**	
	**β**	**p value**	**β**	**p value**
Age	-0.36	<0.01		
Male sex	0.26	<0.01	0.26	<0.01
Body mass index	0.16	0.03	0.16	0.02
Diastolic blood pressure	0.25	<0.01		
Pulse pressure	-0.19	0.01		
Resistive index	-0.40	<0.01	-0.29	<0.01
Estimated GFR	0.35	<0.01	0.25	<0.01
Qualitative urine protein	-0.26	<0.01		
RAAS inhibitors	-0.18	0.02		
Calcium channel blockers	-0.24	<0.01		

GFR, glomerular filtration rate; RAAS, renin-angiotensin-aldosterone system.

### Predictive value of Renal RI for the future development of anemia

Among all 175 patients, we were able to perform follow-up evaluations on 174 (99%) of them. During the follow-up period (median 959 days, range: 7-3595 days), 84 (48%) patients developed anemia (new anemia in 48 patients, further development of anemia in 32 patients, initiation of iron supplementation in 2 patients and initiation of EPO in 2 patients). There was no relationship between the change in hemoglobin level and change in eGFR during the follow-up period. We performed Cox regression analysis in order to determine whether indices of renal damage including renal RI, eGFR and urine protein have potential to predict the future development of anemia. In terms of the results, renal RI per 0.05 rises and the presence of urine protein were independent predictors of the future development of anemia after correcting for sex, diabetes mellitus and baseline anemia (Table 3).

**Table 3:** **Cox proportional hazards regression for the development of anemia**

	**Univariate**		**Multivariate**	
	**Hazard ratio**	**p value**	**Hazard ratio**	**p value**
Age	1.04 (1.01-1.06)	<0.01	1.03 (1.00-1.06)	0.03
Male sex	1.02 (0.61-1.69)	0.95	1.07 (0.63-1.84)	0.80
Diabetes mellitus	1.65 (1.06-2.55)	0.03	1.43 (0.89-2.30)	0.14
Anemia at baseline	1.31 (0.86-2.00)	0.22	0.67 (0.40-1.11)	0.12
Resistive index (per 0.05)	1.30 (1.16-1.46)	<0.01	1.18 (1.02-1.37)	0.03
Estimated GFR	0.99 (0.97-1.00)	0.11	1.00 (0.98-1.01)	0.57
Qualitative urine protein	2.21 (1.44-3.39)	<0.01	1.80 (1.08-3.01)	0.03

GFR, glomerular filtration rate.

**Figure 4:**
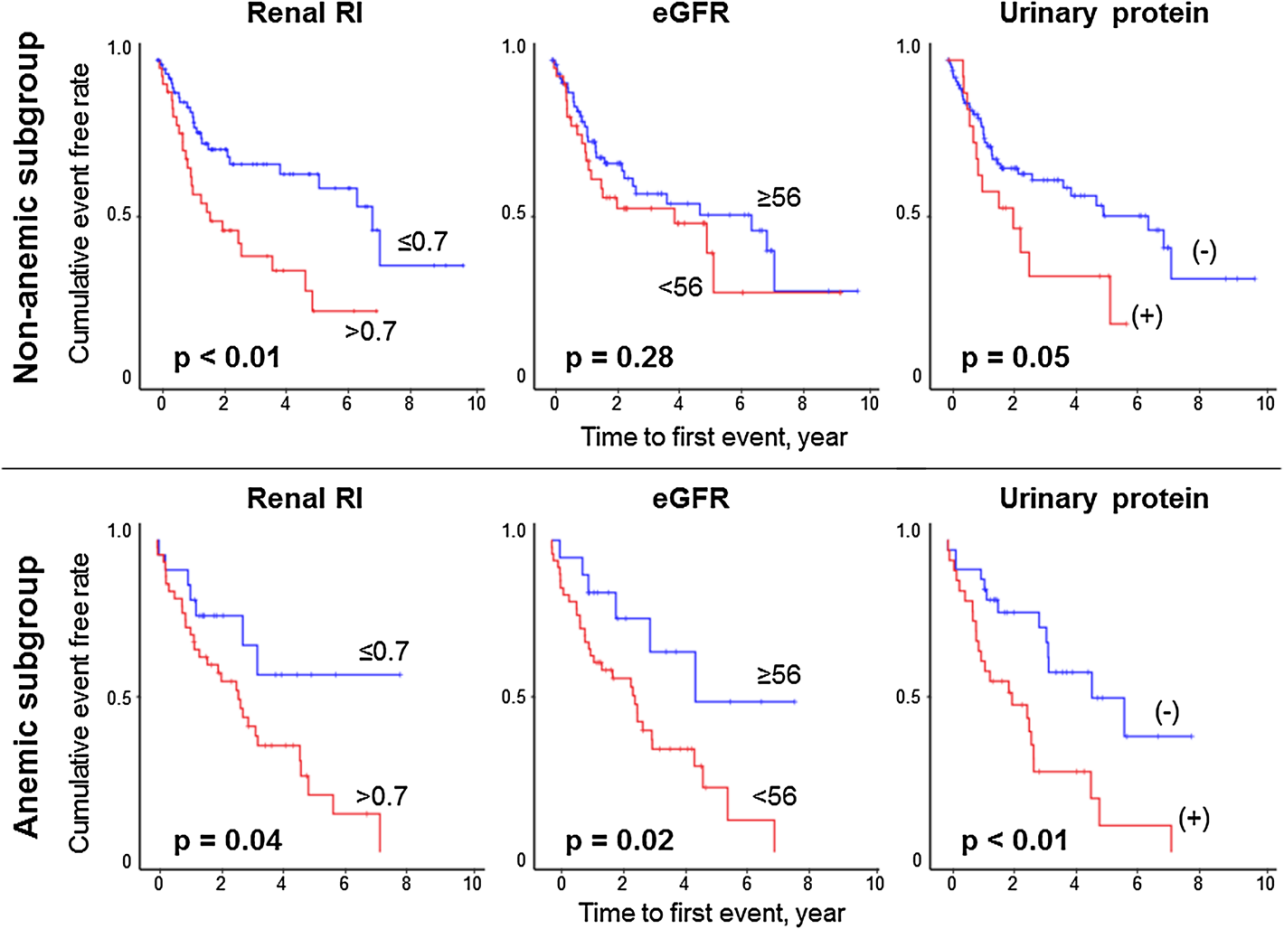
**Kaplan–Meier curves for new anemia in the non-anemic subgroup (top), and further development of anemia in the anemic group (bottom) stratified by median renal RI (>0.70 and ≤0.70, left), median eGFR (<55 and ≥55 ml/min/1.73 m** ^**2**^ **, middle), and the presence or absence of proteinuria (right).** RI, resistive index; eGFR, estimated glomerular filtration rate.

**Figure 5:**
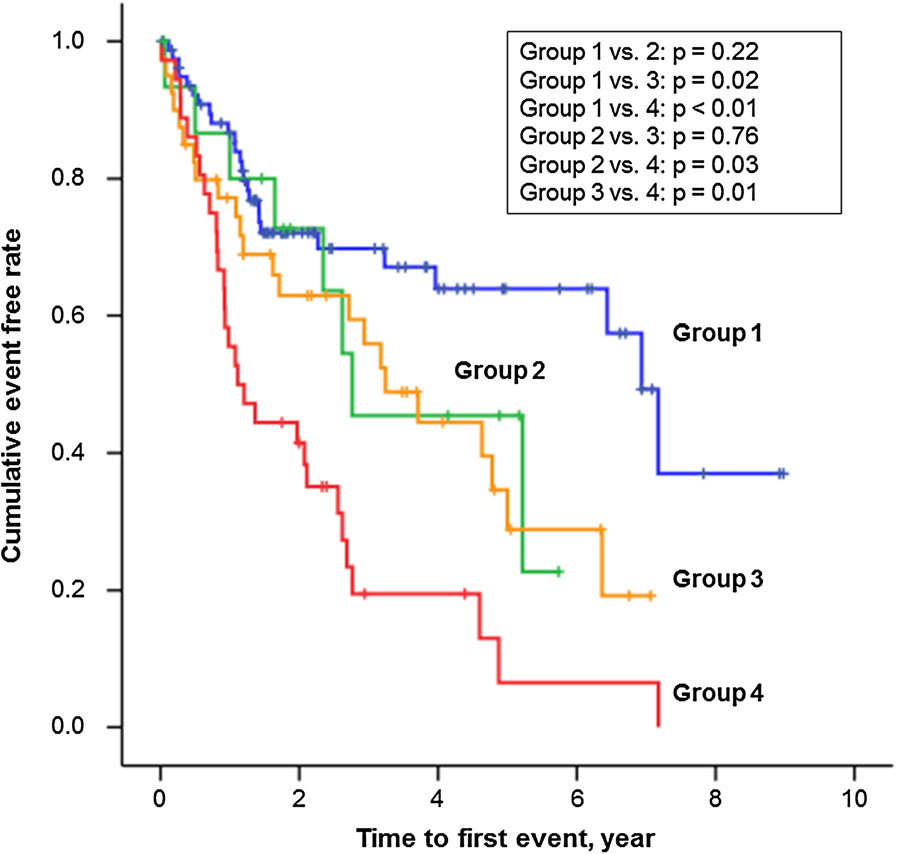
**Kaplan–Meier analysis showing the development of anemia in the 4 groups according to their renal resistive index (RI) values and the presence or absence of proteinuria (group 1: renal RI ≤0.70 and no proteinuria, group 2: renal RI ≤0.70 and proteinuria, group 3: renal RI >0.70 and no proteinuria, and group 4: renal RI >0.70 and proteinuria).**

Kaplan–Meier curves demonstrated that patients with renal RI higher than the median value (0.70) in both the non-anemic and the anemic subgroups had a higher incidence of the future development of anemia than those with renal RI ≤0.70 (Figure 4, left top and left bottom). Patients with eGFR lower than the median value (56 ml/min/1.73 m^2^) only in the anemic subgroup had a higher incidence of the future development of anemia than those with eGFR ≥56 ml/min/1.73 m^2^ (Figure 4, middle top and middle bottom). Patients with the presence of proteinuria had higher incidences of the future development of anemia than those without proteinuria only in the anemic subgroup (Figure 4, right top and right bottom). In addition, when all patients were divided into 4 risk groups according to median renal RI value and the presence or absence of proteinuria (group 1: renal RI ≤0.70 and no proteinuria, group 2: renal RI ≤0.70 and proteinuria, group 3: renal RI >0.70 and no proteinuria, and group 4: renal RI >0.70 and proteinuria), group 1 had a significantly lower risk for the future development of anemia than groups 3 and 4, and group 4 had a significantly higher risk than the other 3 groups (Figure 5).

## Discussion

We demonstrated for the first time that renal RI was independently associated with hemoglobin level in patients with hypertension. Furthermore, high renal RI and the presence of proteinuria predicted the future development of anemia after correcting for confounding factors including age and diabetes mellitus in the present study.

According to Japan’s 2011 National Nutrition Survey, anemia was diagnosed in 8.9% of men and 8.5% of women aged 60-69 in the general population [24]. In the present study, however, the prevalence of anemia was as high as 37% in men and 34% in women in this study population. Renal damage secondary to hypertension is characterized histologically by interstitial fibrosis, arteriosclerosis and glomerular sclerosis [10]. Chronic exposure to high blood pressure initially causes endothelial dysfunction and progressive infiltration of macrophages and T cells to the perivascular interstitium. Interactions of these cells and their cytokines with parenchymal cells cause interstitial fibrosis and tubular insufficiency [7-10]. In recent studies, EPO-producing cells were identified in the renal tubulointerstitium surrounding the central vessels [4,5]. Unfavorable changes in the interstitial microenvironment such as inflammation, oxidative stress and ischemia resulting from hypertension can impair the function of EPO-producing cells. Recently, Souma et al. demonstrated that the phenotypic transition of EPO-producing cells to non-EPO-producing myofibroblasts is modulated by inflammatory molecules, and suggested the connection between anemia and renal fibrosis in CKD in a mouse model [6]. Therefore, the development of CKD secondary to hypertension ought to affect hematopoiesis even in the earlier stages of CKD defined by eGFR. Indeed, there was a significant difference in the hemoglobin levels between patients with renal RI higher than the median value (0.70) and those with renal RI ≤0.7 only in the subgroup of 30 ≤ eGFR <60 ml/min/1.73 m^2^.

We further demonstrated that renal RI and urine protein predicted the future development of anemia. Renal RI reflects the severity of vascular and tubulointerstitial lesions, and has been shown to correlate with an inflammatory state [13-15,25]. We also found that there was no relationship between the change in hemoglobin level and change in eGFR during the follow-up period. These findings may suggest that baseline renal RI and urine protein, rather than worsening glomerular function defined by eGFR, had a greater impact on the development of anemia. Although it would be difficult to identify the underlying mechanisms responsible for the development of anemia precisely, damage of renal interstitial compartments and arteriosclerosis may contribute to the disease process independently of glomerular function, especially in the early stages of CKD defined by eGFR. The causal interrelationship between the progression of CKD and the development of anemia warrants further investigation.

We also demonstrated that the presence of proteinuria is a powerful independent predictor of the future development of anemia. Proteinuria is an important sign of CKD, which can result from hypertension, diabetes mellitus and diseases that cause inflammation in the kidneys. It is recognized that proteins abnormally filtered across the glomerular barrier have intrinsic renal toxicity linked to their over-reabsorption by proximal tubular cells and activation of tubular-dependent pathways of interstitial inflammation and fibrosis [26,27]. Protein overload causes a dose-dependent increase in nuclear factor kappa beta activation in proximal tubular cells that leads to myofibroblast transformation of EPO-producing cells [6,28,29]. We demonstrated that patients with high renal RA and proteinuria had the greatest risk for the future development of anemia. The relationship between proteinuria and renal RI as causal mechanisms underlying the future development of anemia in CKD warrants further investigation. It has been reported that the risk of anemia in patients with diabetes mellitus is approximately two to three times that of the general population with the same level of eGFR due to tubulointerstitial injury secondary to proteinuria and dysglycemia [30]. Diabetes mellitus is commonly found in patients with hypertension and vice versa. In the present study, 52% of patients had diabetes mellitus, and diabetic patients had a higher prevalence of proteinuria than non-diabetic ones. It is difficult to discriminate proteinuria secondary to diabetic nephropathy from that independent of diabetes mellitus per se. However, the presence of proteinuria was independently associated with the future development of anemia after correcting for diabetes mellitus in the present study.

The presence of anemia is known to result in a worse prognosis in terms of both morbidity and mortality. An earlier diagnosis and optimal treatment of anemia would reduce incidence rates of cardiovascular diseases, as well as slow the decline of renal function [31]. Measuring renal RI in addition to conventional markers of renal damage including eGFR and urine protein will help clinicians to decide whether or not the anemia is secondary to renal damage, especially in the early stages of CKD. In addition, high renal RI may aid in alerting them to monitor hemoglobin levels even if patients have no sign of anemia or advanced renal failure on the basis of eGFR.

The limitations of this study include the small sample size and the retrospective nature of the data collection. Renal ultrasonography was performed for the screening of renal artery stenosis and the evaluation of renal arteriosclerosis at the physician’s discretion on the basis of the patients’ age, comorbidity and disease characteristics. Accordingly, the prevalence of coexisting atherosclerotic diseases in patients who were enrolled in the present study can be higher than that of hypertensive patients among the general population. This selection bias may influence the relationship between renal RI and anemia, and the clinical impact of renal RI on the future development of anemia. Renal RI reflects systemic vascular stiffness as well as renal arteriolosclerosis. Indeed, we found a statistically significant positive correlation between renal RI and pulse pressure, one of the surrogate measures of large artery stiffness. Risk factors for systemic atherosclerosis such as inflammation and oxidative stress may affect renal RI and anemia via different pathways, which can confound precise understanding of the causal mechanism underlying the anemia in patients with hypertension and renal damage. However, stepwise multivariate linear regression analyses revealed that renal RI but not pulse pressure was independently associated with hemoglobin level. We were unable to obtain sufficient information to examine precisely the etiology of anemia including iron metabolism, erythropoietin productivity and responsiveness, and inflammation. Iron deficiency is common in patients with CKD. Estrella et al. found that 42.6% of patients who had both anemia and CKD with eGFR between 15 and 59 ml/min/1.73 m^2^ were classified as having functional or absolute iron-deficiency anemia (33.5% and 9.1%, respectively) [32]. Therefore, patients in the anemic group and even in the non-anemic group can have varying degrees of iron deficiency. The causal relationships between iron status and anemia in the hypertensive population, especially those with renal damage, warrant further investigation. Artunc and Risler reported that the correlation between hemoglobin level and EPO concentration was gradually attenuated with increasing stages of CKD and was mostly lost in CKD stages 4 and 5 in an observational study, indicating the presence of more prominent EPO deficiency in advanced CKD [2]. In other words, the pathogenesis of anemia in the early stages of CKD might be heterogeneous in terms of the severity of impairment of EPO productivity and responsiveness. Inflammation also influences EPO efficacy and production as well as renal atherosclerosis. Urine proteins were qualified by using a dipstick test in the present study. Therefore, the potential contribution of microalbuminuria to the pathogenesis of anemia, especially in patients with diabetes, was not investigated. The analysis of markers of tubulointerstitial damage such as beta-2 microglobulins or N-acetylglucosaminidase was also not carried out. Several factors including obesity-related obstructive sleep apnea, current smoking and COPD may contribute to persistent or intermittent hypoxia that lead to red cell production via EPO stimulation. Indeed, body mass index was positively related to hemoglobin level in the present study. However, the presence and severity of sleep apnea and their contribution to hemoglobin level were not assessed in the present study. Finally, follow-up data of renal RI, urine protein, and changes in nutritional status and medication were not included in our outcome evaluation.

## Conclusion

In conclusion, renal RI correlated directly with hemoglobin levels and contributed to the development of anemia independently of glomerular function. The measurement of renal RI in patients with hypertension can be useful for elucidating the pathogenesis of anemia and for inferring its potential risk.

## Abbreviations

CKD: Chronic kidney disease
EPO: Erythropoietin
RI: Resistive index
GFR: Glomerular filtration rate
eGFR: Estimated glomerular filtration rate
SCr: Serum creatinine
RAAS: Renin-angiotensin-aldosterone system
